# Correlation between Genomic Variants and Worldwide COVID-19 Epidemiology

**DOI:** 10.3390/jpm14060579

**Published:** 2024-05-28

**Authors:** Ana Caroline Alves da Costa, Laura Patrícia Albarello Gellen, Marianne Rodrigues Fernandes, Rita de Cássia Calderaro Coelho, Natasha Monte, Francisco Cezar Aquino de Moraes, Maria Clara Leite Calderaro, Lilian Marques de Freitas, Juliana Aires Matos, Thamara Fernanda da Silva Fernandes, Kaio Evandro Cardoso Aguiar, Lui Wallacy Morikawa Souza Vinagre, Sidney Emanuel Batista dos Santos, Ney Pereira Carneiro dos Santos

**Affiliations:** 1 Oncology Research Center, Federal University of Pará, Belém 66073-005, PA, Brazil; carolinecostaodonto19@gmail.com (A.C.A.d.C.); laura.patricia.agellen@hotmail.com (L.P.A.G.); fernandesmr@yahoo.com.br (M.R.F.); rccalderarocoelho@gmail.com (R.d.C.C.C.); ntshmonte@gmail.com (N.M.); francisco.cezar2205@gmail.com (F.C.A.d.M.); mariaclaracalderaro@gmail.com (M.C.L.C.); lilianmarques@gmail.com (L.M.d.F.); juliana.matos@ics.ufpa.br (J.A.M.); kaio.evandro@hotmail.com (K.E.C.A.); sidneysan-tos@ufpa.br (S.E.B.d.S.); 2 Ophir Loyola Hospital, Pará State Departament of Health, Belém 66063-240, PA, Brazil; 3 Laboratory of Human and Medical Genetics, Institute of Biological Science, Federal University of Pará, Belém 66077-830, PA, Brazil

**Keywords:** SARS-CoV-2, genetic risk variant, susceptibility, mortality, epidemiology

## Abstract

COVID-19 is a systemic disease caused by the etiologic agent SARS-CoV-2, first reported in Hubei Province in Wuhan, China, in late 2019. The SARS-CoV-2 virus has evolved over time with distinct transmissibility subvariants from ancestral lineages. The clinical manifestations of the disease vary according to their severity and can range from asymptomatic to severe. Due to the rapid evolution to a pandemic, epidemiological studies have become essential to understand and effectively combat COVID-19, as the incidence and mortality of this disease vary between territories and populations. This study correlated epidemiological data on the incidence and mortality of COVID-19 with frequencies of important SNPs in GWAS studies associated with the susceptibility and mortality of this disease in different populations. Our results indicated significant correlations for 11 genetic variants (rs117169628, rs2547438, rs2271616, rs12610495, rs12046291, rs35705950, rs2176724, rs10774671, rs1073165, rs4804803 and rs7528026). Of these 11 variants, 7 (rs12046291, rs117169628, rs1073165, rs2547438, rs2271616, rs12610495 and rs35705950) were positively correlated with the incidence rate, these variants were more frequent in EUR populations, suggesting that this population is more susceptible to COVID-19. The rs2176724 variant was inversely related to incidence rates; therefore, the higher the frequency of the allele is, the lower the incidence rate. This variant was more frequent in the AFR population, which suggests a protective factor against SARS-CoV-2 infection in this population. The variants rs10774671, rs4804803, and rs7528026 showed a significant relationship with mortality rates. SNPs rs10774671 and rs4804803 were inversely related to mortality rates and are more frequently present in the AFR population. The rs7528026 variant, which is more frequent in the AMR population, was positively related to mortality rates. The study has the potential to identify and correlate the genetic profile with epidemiological data, identify populations that are more susceptible to severe forms of COVID-19, and relate them to incidence and mortality.

## 1. Introduction

COVID-19 is a severe acute respiratory infection with systemic manifestation that was first reported in early December 2019 in Wuhan, Hubei Province, China [1]. SARS-CoV-2 is a virus of the genus Betacoronavirus that has similarities to SARS-CoV in tissue tropism, genome structure, and viral pathogenesis. SARS-CoV-2 is a shrouded, positive-sense single-stranded RNA virus. The genomic sequence of SARS-CoV-2 shares about 80% of the sequence identity with SARS-CoV and about 50% with MERS-CoV. However, despite the similarities with SARS-CoV and MERS-CoV, SARS-CoV-2 has been shown to be more transmissible and the diversity of immune responses of its hosts is still poorly understood [2].

The pandemic caused by SARS-CoV-2 infection has caused severe disruption to health systems and global economies, with significant variations in incidence and mortality rates. Critical COVID-19 develops in less than 10% of individuals infected with SARS-CoV-2; however, studies point to a high genetic risk (greater than 10%) in individuals who may or may not have clinical risk factors [3]. The global public health response to SARS-CoV-2 includes different degrees of interventions, according to the containment policies adopted and socioeconomic conditions [4].

The SARS-CoV-2 virus has evolved over time with subvariants of transmissibility distinct from ancestral lineages, predominant in successive epidemic waves. The clinical manifestations of the disease vary according to their severity and can range from asymptomatic to severe [5,6,7]. At the beginning of the pandemic, advanced age and comorbidities were consolidated as important risk factors [8]. However, in isolation, these factors cannot explain the clinical variability of the disease observed among individuals. Studies in genomics can potentially explain the greater predisposition to severe forms of COVID-19 and thus contribute to the discovery of related pathways and their probable therapeutic use [9]. 

As of 1 September 2023, a total of 539,873,221 cases of COVID-19 have been confirmed for the populations analyzed in this study: Europe (EUR), Africa (AFR), East Asia (EAS), South Asia (SAS), and the Americas (AMR). Data obtained by the World Health Organization (WHO) indicate that the population with the highest incidence of the disease is EUR (51.10%), followed by the AMR population (37.78%). On the other hand, the population of African origin had the lowest incidence rate (1.76%). Of the total number of cases registered, 6,814,976 individuals died, of which the highest mortality rate was observed in individuals from the American population (47%) and the lowest rate was observed in the AFR population of origin (2.83%) [10]. 

In this context, epidemiological studies that correlate genetic variants and global epidemiology are extremely important, as they help in the identification of genes and pathways related to mortality and COVID-19 susceptibility, establishing the possible relationship of genetic variants with epidemiological rates. Therefore, our study aims to correlate epidemiological data on COVID-19 incidence and mortality worldwide with the frequency of important variants related to disease susceptibility and mortality. 

## 2. Materials and Methods

### 2.1. Determination of SNPs

For the SNPs associated with COVID-19 selection that were evaluated in the study, articles with variants associated with susceptibility and COVID-19 mortality were selected. For this, articles in English were selected from the Medline/PubMed databases, according to the keywords COVID-19, SARS-CoV-2, GWAS, risk and susceptibility. Inclusion criteria were based on the selection of studies of genomic variants associated with susceptibility and severity to COVID-19 in the period 2019 to 2023. 

**Figure 1:**
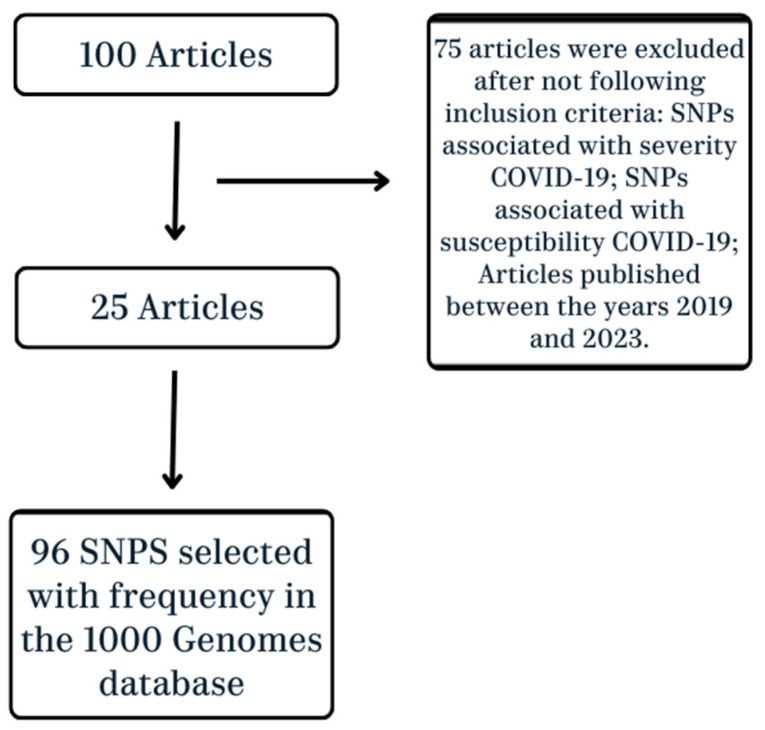
SNPs selected flowchart.

A total of 100 articles were found, of which, those that did not contain variants associated with susceptibility or more severe forms of COVID-19 were excluded, leaving only 25 articles (Supplementary Tables). After reading the studies, we selected 96 variants related to susceptibility or severity to COVID-19 that had their frequency described in the 1000 Genomes database (Figure 1).

### 2.2. Epidemiological and Genetic Data

The incidence and mortality rates of COVID-19 for the population were obtained from the World Health Organization platform for Coronavirus (COVID-19), available on the WHO Coronavirus (COVID-19) Dashboard|WHO Coronavirus (COVID-19) Dashboard with Vaccination Data [10].

In the 1000 Genomes Project, the allele frequencies of genetic variants in the following continental populations were evaluated: Europe (EUR), Africa (AFR), East Asia (EAS), South Asia (SAS) and Americas (AMR). 

We compared WHO populations with continental populations available in phase 3 of the 1000 Genomes URL www.1000genomes.org database (accessed on 20 August 2023) [11]. According to the Consortium of the 1000 Genomes Project, populations are grouped by the predominant component of ancestry, so our study correlated the epidemiological and genetic data of the populations of EUR, AFR, EAS, SAS, and AMR. For the statistical analyses, the East and South Asian populations were grouped due to their genetic similarities.

### 2.3. Statistical Analysis

The incidence and severity of COVID-19, as well as the frequencies of variant alleles, were assessed using Pearson’s correlation test. The data were evaluated with the groups described above (AFR, AMR, EUR, EAS, and SAS), using the “color.test” function of the “stats” package of the R program. After this procedure, r, r2 and *p*-values were obtained. All graphs were created using the “ggplot2” graphics package. Results lower than *p* ≤ 0.05 were considered statistically significant.

## 3. Results

**Figure 2:**
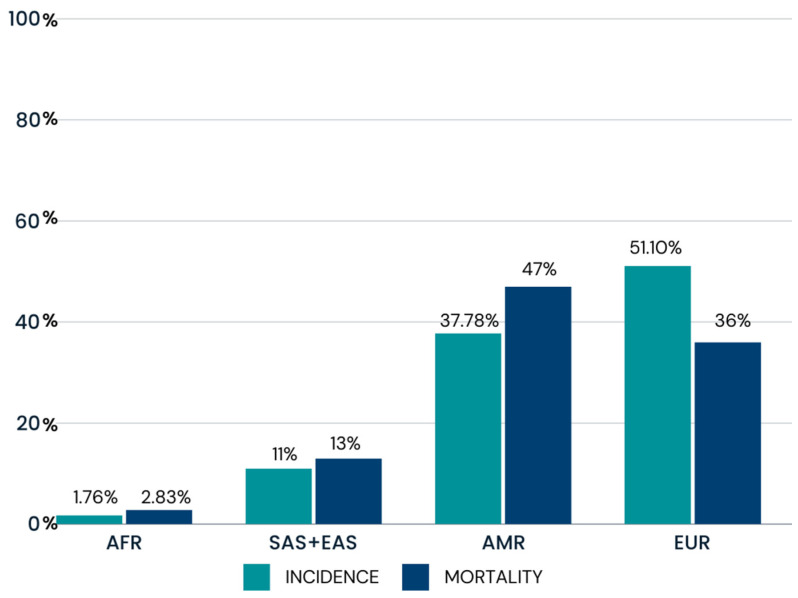
COVID-19 incidence and mortality in 2023 from World Health Organization registries, in continental populations EUR, AFR, EAS, SAS, and AMR. AFR: African population; AMR: American population; EUR: European population; SAS: South Asian population; EAS: East Asian population.

A total of 539,873,221 cases of COVID-19 were confirmed worldwide as of 1 September 2023 for the populations analyzed in this study. Of these, 51.10% of the cases were in EUR, 37.78% in AMR, 11.33% in SAS and EAS and 1.76% in AFR. Of the total cases, 6,814,976 individuals died, 47% in AMR, 36% in EUR, 13% in SAS and EAS, and 2.83% in Africa. Figure 2 shows the incidence and mortality rates from COVID-19 in 2023. The EUR population had the highest incidence rates; however, the AMR population stood out with the highest number of deaths [10]. 

A total of 96 variants (Supplementary Tables) associated with susceptibility and severity to COVID-19 were selected as described in Figure 1. Among these 96 analyzed variants, 11 (rs117169628, rs2547438, rs2271616, rs12610495, rs12046291, rs35705950, rs2176724, rs10774671, rs1073165, rs4804803 and rs7528026) were significant in Pearson’s correlation analysis and for epidemiological data (Table 1). Of these variants, two (rs1073165 and rs2176724) are in intergenic regions, these regions, despite not being encoded by proteins, regulate the expression of nearby genes (Intergenic Regions (genome.gov/, accessed on 1 November 2023)) [12]. 

**Figure 3:**
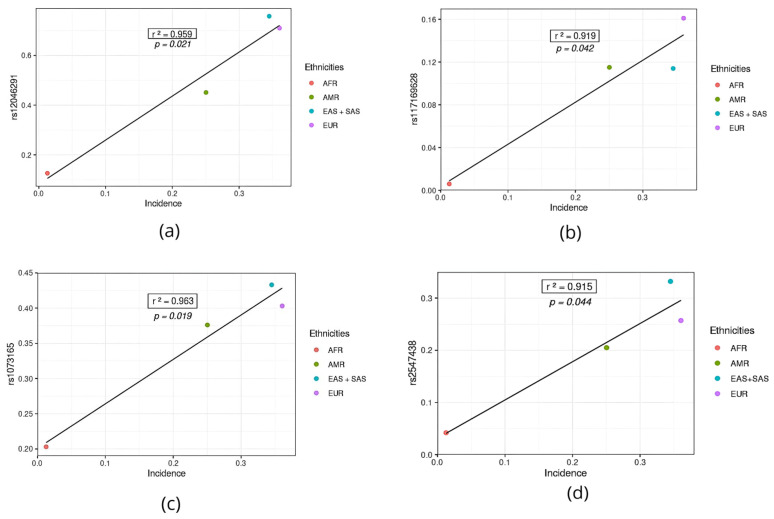

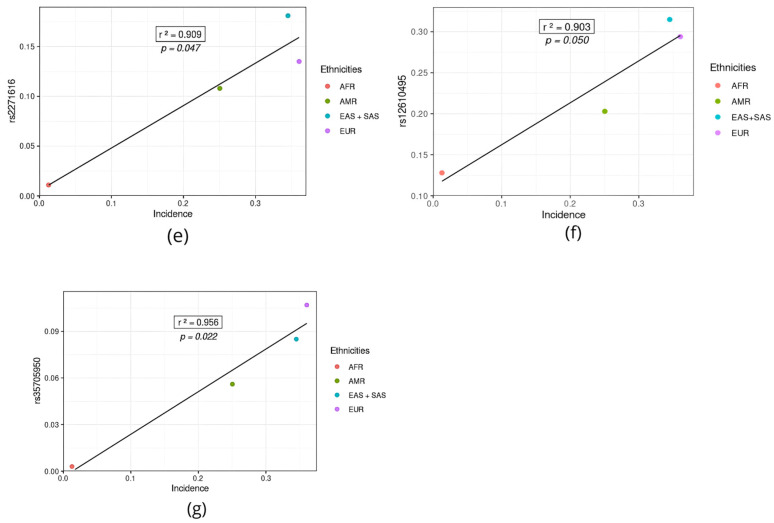
SNPs positively associated with COVID-19 incidence in different populations. (**a**) SNP rs12046291. (**b**) SNP rs117169628. (**c**) SNP rs1073165. (**d**) SNP rs2547438. (**e**) SNP rs2271616. (**f**) SNP rs12610495. (**g**) SNP rs35705950. AFR: African population; AMR: American population; EUR: European population; SAS: South Asian population; EAS: East Asian population.

Of these 11, 7 (rs12046291, rs117169628, rs1073165, rs2547438, rs2271616, rs12610495 and rs35705950) were positively correlated with the incidence rate (Figure 3). The estimated data denote that the higher the frequency of the variant allele of these variants, the higher the numbers of new cases estimated in the world’s populations. We observed that all variants had a higher frequency in the population of European origin (EUR) and lower frequency in Africans. This analysis is confirmed by numerical data on global epidemiology (WHO data). According to our analyses, the European population is more susceptible to COVID-19 infection.

**Figure 4:**
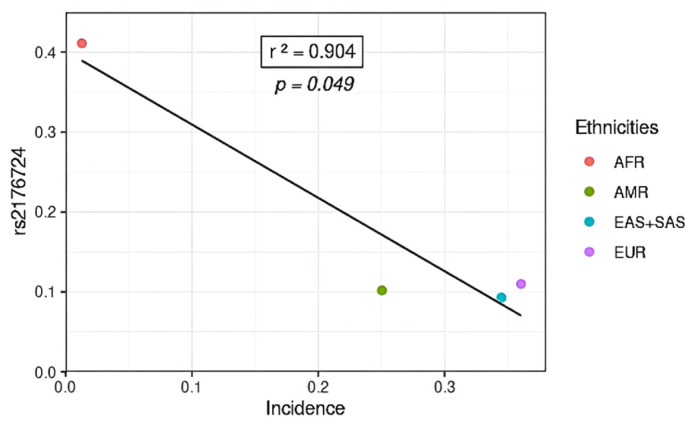
SNP inversely associated with COVID-19 incidence in different populations. AFR: African population; AMR: American population; EUR: European population; SAS: South Asian population; EAS: East Asian population.

In contrast, one variant (rs2176724) was inversely correlated with incidence rates, so the higher the frequency of the variant allele, the lower the incidence rate (Figure 4). The variant was more frequent in the African population (AFR). In the global numerical data, the African population had the lowest incidence rates for COVID-19 when compared to other populations (Figure 2). In view of this, it is possible to suggest the protective factor of these variants against SARS-CoV-2 infection in African ancestry.

**Figure 5:**
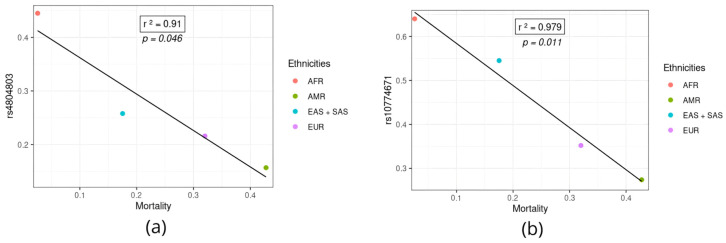
SNPs inversely associated with COVID-19 mortality rates in different populations. (**a**) SNP rs4804803. (**b**) SNP rs10774671. AFR: African population; AMR: American population; EUR: European population; SAS: South Asian population; EAS: East Asian population.

Figure 5 shows the two variants (rs10774671 and rs4804803) that showed a statistically significant correlation with the mortality rate. Both were inversely correlated with mortality rates, so that the higher the frequency of the variant allele, the lower the mortality rate. The two variants are more frequent in the African population and may be a protective factor against the disease caused by SARS-CoV-2 infection. This fact is confirmed by the graph in Figure 2, which shows the lowest mortality rates in the African population.

**Figure 6:**
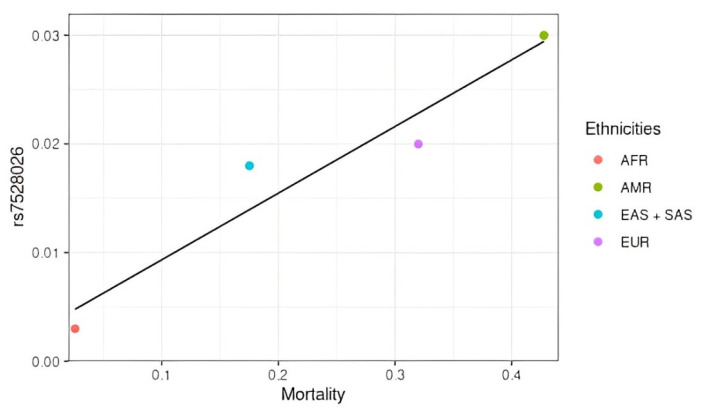
SNPs positively associated with COVID-19 mortality in different populations. AFR: African population; AMR: American population; EUR: European population; SAS: South Asian population; EAS: East Asian population.

rs7528026 was positively correlated with mortality rates (Figure 6), i.e., the higher the frequency of the variant allele, the higher the number of deaths. The highest mortality rates were in the American population, and this analysis is justified by the world’s epidemiology.

## 4. Discussion

COVID-19 incidence and mortality rates vary significantly by ethnicity, showing a wide fluctuation in epidemiological rates (WHO data). Our results demonstrated that the most genetically important variants related to the incidence of COVID-19 are positively correlated with populations of European origin, this has been demonstrated in epidemiological data from the World Health Organization. Regarding mortality, global epidemiological data show that Latino populations have higher mortality rates for COVID-19. Three variants (rs10774671, rs4804803, and rs7528026) were important to relate genetic influence to the estimated data for the AMR population, two (rs10774671 and rs4804803) were inversely related to mortality, and one (rs7528026) was positively related.

This study correlated epidemiological data on COVID-19 incidence and mortality with frequencies of important SNPs in GWA studies associated with the susceptibility and mortality of this disease in different populations. Our results indicated significant correlations for 11 genetic variants (rs117169628, rs2547438, rs2271616, rs12610495, rs12046291, rs35705950, rs2176724, rs10774671, rs1073165, rs4804803 and rs7528026), 2 of them in intergenic regions and 9 in 9 genes (*C3*, *SLC6A20*, *DPP9*, *MUC5B*, *OAS1*, *CD209*, *JAK1*, *SLC22A31* and *TRIM46*) with the incidence and mortality rates of the disease. Our results show that variants related to high incidence are more frequent in the European population and less frequent in the African population.

Of the variants studied, two (rs1073165 and rs2176724) are in intergenic regions. The rs1073165 SNP was associated with the incidence of COVID-19 and the rs2176724 variant was shown to be an important protective factor against infection (incidence), being inversely related to the incidence of the disease, we observed a higher frequency of this variant in the African population. This analysis is confirmed with data from World Epidemiology. In addition, we identified important localized variants in genes responsible for regulating the immune system, such as *JAK1*, *DPP9*, *MUC5B*, *CD209*, *OAS1*, and *TRIM46*. In our results, the *JAK1*, *DPP9*, and *MUC5B* genes had their variants (rs12046291, rs12610495, and rs3570595) correlated with the risk of developing the disease/incidence and the *CD209*, *OAS1,* and *TRIM46 genes* had their variants (rs4804803, rs10774671 and rs7528026) associated with mortality. 

The *JAK1* gene is a gene that encodes a protein that is inhibited by baricitinib and other inhibitors of *JAK*—the intracellular signaling kinase [13]. The literature has revealed a significant positive association between gene expression and disease severity [7,9,14,15,16]. In our study, we found a positive correlation in the rs12046291 variant present in this gene with COVID-19 incidence rates, this variant was more frequent in the European population. In addition, the rs12046291 variant had significant associations with critical COVID-19 in GWAs studies [13]. This suggests that inhibiting the signaling of this gene may be an effective therapy for severe COVID-19.

*DPP9* belongs to the serine protease family *S9B/DPPIV* and is responsible for regulating the inflammatory response. A recent study demonstrated that patients with severe stage COVID-19 expressed higher levels of *DPP9* compared to healthy control cases [8]. In our analyses, the variant (rs12610495) located in this gene was associated with COVID-19 incidence, this SNP was found more frequently in the EUR population, which reinforces that this population is more susceptible to SARS-CoV-2 infection. 

Mucins belong to the first defense against pathogens in the airways and are essential for mucociliary correction; high expression of *MUC5B* may be related to worse prognosis of SARS-CoV-2 infection [8,14,17]. The rs35705950 SNP present in this gene is described as an important variant associated with hospitalization and idiopathic pulmonary fibrosis [14]. In our results, the rs35705950 variant was more frequently present in the European population and was positively related to COVID-19 incidence rates. 

*SLC620* is a sodium transporter that can functionally interact with angiotensin-converting enzyme 2, the cell surface receptor of SARS-CoV-2 [18,19]. The rs2271616 found in our study suggests the potential involvement of this variant in the gene *SLC6A20’s* susceptibility to COVID-19, which is represented by the incidence of this disease. The same observation was made in the rs11719628 variant found in the *SLC22A31 gene*, this gene is part of the transmembrane transporter family and the variant described in this gene (rs11719628) was associated with severity to COVID-19 in genomic sequencing studies conducted on COVID-19 patients [7,20,21]. 

In our study, we identified genetic variants associated with mortality and located in genes that are fundamental for the regulation of immune responses, an example is rs10774671 present in the gene Oligoadenilato Sintetase 1 (*OAS1*). The genetic variants present in this gene have been strongly related to susceptibility to viral infections such as SARS-CoV-2 [20,22,23]. In our study, the mutant variant present in this gene (rs10774671) was inversely associated with COVID-19 mortality, which suggests that this variant is a protective factor against COVID-19, this SNP was more frequent in the AFR population.

*CD209* is described as an important encoder of C-type lectin, which is also related to the recognition of several viral pathogens including the SARS virus [9,24,25]. In our findings, rs4804803 demonstrated an inverse relationship with mortality rates from the disease, which is mainly present in the African population, suggesting a protective factor against SARS-CoV-2 infection in this population. This fact reinforces what we observed in the epidemiological data of the WHO (Figure 2), where it is possible to observe that the AFR population had lower mortality rates from the disease. 

The rs7528026 located in the *TRIM46* gene was positively associated with mortality, this variant was more frequent in the AMR population. This gene is an important signaler of interferon gamma pathways and cytokine signaling in the immune system, and is associated with inflammatory bowel disease, lung cancer chemoresistance, and susceptibility to severe influenza virus infections [26,27,28]. In addition, the rs7528026 variant was significantly associated with critical forms of COVID-19 in GWAs studies [13].

COVID-19 incidence and mortality rates vary significantly by ethnicity, showing a wide fluctuation in epidemiological rates (WHO data). This study had limitations in relation to information on certain ethnic groups, such as Africans, due to the lack of homogeneity in socioeconomic levels in the populations analyzed. Despite these limitations, this study contributes important findings to the literature and may help to choose genetic markers that are more globally homogeneous in relation to COVID-19 incidence and prognosis.

Our results demonstrated that the most important variants directly correlated with the incidence/susceptibility of COVID-19 are more frequently present in populations of European origin, which may suggest that this population is potentially more susceptible to infection by the SARS-COV-2 virus. Epidemiological data have shown that the highest mortality rates worldwide were found in populations of Latino origin, our results found three important variants related to the immune response that may contribute in part to the increase in case fatality rates in these populations. 

The present study may contribute to a homogenization of data from studies with GWAs around the world, since we often find different results between these variants and their correlation with epidemiological data regarding susceptibility/incidence and prognosis/mortality of COVID-19.

## 5. Conclusions

Our study demonstrated that 11 SNPs (rs117169628, rs2547438, rs2271616, rs12610495, rs12046291, rs35705950, rs2176724, rs10774671, rs1073165, rs4804803 and rs7528026) were correlated with epidemiological data in different ethnic groups. Seven SNPs (rs12046291, rs117169628, rs1073165, rs2547438, rs2271616, rs12610495 and rs35705950) were positively correlated with the incidence rate. One variant was inversely associated with incidence rates (rs2176724). Two variants (rs10774671 and rs4804803) were inversely correlated with mortality rates. In contrast, one variant (rs7528026) was positively related to mortality rates.

Our study has the potential to identify and correlate the genetic profile with epidemiological data, to identify populations that are more susceptible to severe forms of COVID-19 and relate them to incidence and mortality. This study shows promise for the implementation of personalized strategies for molecular screening of COVID-19 in diverse populations around the world, a fact that can contribute to the identification of susceptible individuals and more effectively manage the evolution of the disease.

## Figures and Tables

**Table 1:** Description of variants that have been significantly correlated with COVID-19 incidence and/or mortality in continental (African (AFR), American (AMR), European (EUR), and East Asian (EAS) + South Asian (SAS)) populations described in the 1000 Genomes database and *p*-values.

Gene	SNP ID/Association	Most Severe Consequence	Alleles	Ancestral	Clinical Impact	*p* Value	Frequences			
							AFR	AMR	EUR	SAS + EAS
*C3*	rs2547438/ Incidence	Intron Variant	T/C/G	G	Benign	0.0436	0.042	0.205	0.257	0.166
*SLC6A20*	rs2271616/ Incidence	5′ UTR Variant	G/T	G	Benign	0.0497	0.011	0.108	0.135	0.09
*DPP9*	rs12610495/ Incidence	Intron Variant	A/G/T	A	Not Reported in ClinVar	0.0498	0.128	0.203	0.294	0.157
*MUC5B *	rs35705950/ Incidence	None	G/A/T	G	Benign; risk factor	0.0221	0.003	0.056	0.107	0.0425
- *	rs2176724/ Incidence	Intergenic Variant	G/A	G	Not Reported in ClinVar	0.0494	0.411	0.102	0.11	0.046
*OAS1*	rs10774671/ Mortality	Splice Acceptor Variant	G/A/C	G	Benign	0.0105	0.64	0.274	0.352	0.272
*CD209*	rs4804803/ Mortality	2 KB Upstream Variant	A/G	G	protective; risk factor	0.046	0.445	0.157	0.216	0.129
*JAK1*	rs12046291/ Incidence	Intron Variant	A/G	A	Not Reported in ClinVar	0.021	0.126	0.451	0.710	0.379
*SLC22A31*	rs117169628/ Incidence	Missense Variant	G/A	G	Benign	0.042	0.006	0.115	0.161	0.057
- *	rs1073165/ Incidence	Intergenic Variant	A/G	A	Not Reported in ClinVar	0.019	0.203	0.376	0.403	0.2165
*TRIM46*	rs7528026/ Mortality	Intron Variant	G/A	G	Not Reported in ClinVar	0.049	0.003	0.030	0.020	0.009

- *: No annotation.

## Data Availability

All relevant data will be shared as Supplementary Materials if the manuscript is accepted for publication.

## Supplementary Materials

The following supporting information can be downloaded at https://www.mdpi.com/article/10.3390/jpm14060579/s1: Table S1: SNPs correlated with the incidence of COVID-19 in different populations. Table S2: SNPs that correlated with COVID-19 mortality in different populations. Table S3: 25 studies included in the review [29,30,31,32,33,34,35,36,37,38,39,40,41,42,43,44,45,46,47].
