# Supplementary material for: Correlation between Genomic Variants and Worldwide COVID-19 Epidemiology
Source: J Pers Med. 2024 May 28;14(6):579. doi: 10.3390/jpm14060579 (PMC11204818; doi:10.3390/jpm14060579)
Supplement: Supplementary file 1 [file jpm-14-00579-s001.zip › jpm-2999386-supplementary.pdf]

Supplementary Table S1. SNPs correlated with the incidence of COVID-19 in different populations.

GENE	SNP ID	Freq. AFR	Inc AFR	Freq. AMR	Inc AMR	Freq. EAS+SAS	Inc EAS+SAS	Freq. EUR	Inc EUR	R	R^2	*P*_valor
STXBP5-AS1	**rs117928001**	0.003	0.0128	0.012	0.2504	0.026	0.345	0.046	0.3605	0.835	0.697	0.165
LOC105373891	**rs34011564**	0.123	0.0128	0.225	0.2504	0.208	0.345	0.237	0.3605	0.929	0.864	0.0706
CPQ	**rs4735444**	0.368	0.0128	0.271	0.2504	0.938	0.345	0.208	0.3605	0.242	0.0583	0.758
-	**rs117217714**	0	0.0128	0.006	0.2504	0.008	0.345	0.006	0.3605	0.949	0.9	0.0514
-	**rs2176724**	0.411	0.0128	0.102	0.2504	0.093	0.345	0.11	0.3605	-0.951	0.904	0.0494
PCDH15	**rs9804218**	0.461	0.0128	0.514	0.2504	0.612	0.345	0.693	0.3605	0.874	0.765	0.126
CLUAP1	**rs2301762**	0	0.0128	0.071	0.2504	0.208	0.345	0.052	0.3605	0.658	0.434	0.342
C3	**rs2547438**	0.042	0.0128	0.205	0.2504	0.332	0.345	0.257	0.3605	0.956	0.915	0.0436
C3	**rs2250656**	0.184	0.0128	0.255	0.2504	0.48	0.345	0.297	0.3605	0.73	0.533	0.27
THBD	**rs1042580**	0.201	0.0128	0.291	0.2504	0.683	0.345	0.368	0.3605	0.694	0.482	0.306
CFH	**rs800292**	0.791	0.0128	0.419	0.2504	0.75	0.345	0.26	0.3605	-0.565	0.319	0.435
CFHR1	**rs414628**	0.326	0.0128	0.507	0.2504	0.897	0.345	0.443	0.3605	0.622	0.386	0.378
-	**rs73060484**	0.115	0.0128	0.236	0.2504	0.369	0.345	0.077	0.3605	0.356	0.127	0.644
-	**rs77578623**	0.114	0.0128	0.236	0.2504	0.372	0.345	0.078	0.3605	0.363	0.132	0.637
-	**rs74417002**	0.067	0.0128	0.024	0.2504	0.033	0.345	0.026	0.3605	-0.902	0.813	0.0982
-	**rs73070529**	0.218	0.0128	0.236	0.2504	0.281	0.345	0.058	0.3605	-0.263	0.0692	0.737
LINC01827	**rs113892140**	0.256	0.0128	0.238	0.2504	0.279	0.345	0.046	0.3605	-0.439	0.192	0.561
DNAH7	**rs183712207**	0.001	0.0128	0.009	0.2504	0	0.345	0.009	0.3605	0.384	0.147	0.616
DNAH7	**rs191631470**	0.001	0.0128	0.009	0.2504	0	0.345	0.014	0.3605	0.467	0.218	0.533
STBP5	**rs116898161**	0.002	0.0128	0.013	0.2504	0.026	0.345	0.041	0.3605	0.886	0.784	0.114
-	**rs55986907**	0.123	0.0128	0.337	0.2504	0.533	0.345	0.284	0.3605	0.774	0.598	0.226
CPQ	**rs1431889**	0.299	0.0128	0.262	0.2504	0.938	0.345	0.209	0.3605	0.332	0.11	0.668
CPQ	**rs2874140**	0.419	0.0128	0.287	0.2504	0.955	0.345	0.21	0.3605	0.181	0.0328	0.819
CPQ	**rs7007951**	0.348	0.0128	0.277	0.2504	0.935	0.345	0.2	0.3605	0.262	0.0685	0.738
CPQ	**rs920576**	0.322	0.0128	0.284	0.2504	0.961	0.345	0.221	0.3605	0.322	0.103	0.678
-	**rs60811869**	0.03	0.0128	0.019	0.2504	0.019	0.345	0.03	0.3605	-0.4	0.16	0.6
LZTFL1	**rs10490770**	0.004	0.0128	0.043	0.2504	0.301	0.345	0.081	0.3605	0.625	0.39	0.375
-	**rs115679256**	0.002	0.0128	0.02	0.2504	0.01	0.345	0.046	0.3605	0.67	0.449	0.33
LOC107986082	**rs17763742**	0.004	0.0128	0.042	0.2504	0.306	0.345	0.081	0.3605	0.62	0.385	0.38
FYCO1	**rs41289622**	0.005	0.0128	0.059	0.2504	0.365	0.345	0.122	0.3605	0.671	0.45	0.329
LOC105377067	**rs115102354**	0.002	0.0128	0.019	0.2504	0.024	0.345	0.048	0.3605	0.858	0.736	0.142
-	**rs10813976**	0.11	0.0128	0.192	0.2504	0.432	0.345	0.196	0.3605	0.658	0.433	0.342
ARHGAP27	**rs1230082**	0.53	0.0128	0.373	0.2504	0.918	0.345	0.303	0.3605	0.0887	0.00788	0.911
-	**rs77127536**	0.174	0.0128	0.141	0.2504	0.494	0.345	0.181	0.3605	0.421	0.177	0.579
IFNAR2	**rs17860169**	0.194	0.0128	0.451	0.2504	1.098	0.345	0.333	0.3605	0.427	0.183	0.573
FYCO1	**rs35477280**	0.005	0.0128	0.058	0.2504	0.378	0.345	0.12	0.3605	0.658	0.433	0.342
SLC6A20	**rs2271616**	0.011	0.0128	0.108	0.2504	0.183	0.345	0.135	0.3605	0.95	0.903	0.0497
OAS1	**rs10774671**	0.64	0.0128	0.274	0.2504	0.545	0.345	0.352	0.3605	-0.576	0.332	0.424
RAVER1	**rs74956615**	0.002	0.0128	0.026	0.2504	0.02	0.345	0.03	0.3605	0.9	0.811	0.0997
TYK2	**rs34536443**	0.002	0.0128	0.02	0.2504	0.006	0.345	0.029	0.3605	0.624	0.389	0.376
PLEKHA4	**rs4801778**	0.16	0.0128	0.105	0.2504	0.166	0.345	0.192	0.3605	0.224	0.0504	0.776
PPP1R15A	**rs11541192**	0.247	0.0128	0.102	0.2504	0.185	0.345	0.185	0.3605	-0.52	0.271	0.48
DPP9	**rs2109069**	0.196	0.0128	0.219	0.2504	0.326	0.345	0.321	0.3605	0.87	0.757	0.13
FOXP4-AS1	**rs1886814**	0.083	0.0128	0.197	0.2504	0.494	0.345	0.043	0.3605	0.392	0.154	0.608
FOXP4-AS1	**rs7741164**	0.089	0.0128	0.2	0.2504	0.488	0.345	0.032	0.3605	0.36	0.13	0.64

KANSL1	**rs1819040**	0.062	0.0128	0.16	0.2504	0.065	0.345	0.242	0.3605	0.54	0.292	0.46
CD209	**rs4804803**	0.445	0.0128	0.157	0.2504	0.258	0.345	0.216	0.3605	-0.813	0.661	0.187
IFNAR2	**rs13050728**	0.194	0.0128	0.451	0.2504	1.095	0.345	0.333	0.3605	0.427	0.183	0.573
OAS1	**rs4767027**	0.011	0.0128	0.203	0.2504	0.538	0.345	0.345	0.3605	0.888	0.789	0.112
DPP9	**rs12610495**	0.128	0.0128	0.203	0.2504	0.315	0.345	0.294	0.3605	0.95	0.903	0.0498
TYK2	**rs11085727**	0.094	0.0128	0.258	0.2504	0.741	0.345	0.27	0.3605	0.661	0.437	0.339
HLA-DPA1	**rs2071351**	0.604	0.0128	0.281	0.2504	1.049	0.345	0.186	0.3605	0.427	0.182	0.573
ACE2	**rs190509934**	0.006	0.0128	0.006	0.2504	0.033	0.345	0.003	0.3605	0.357	0.128	0.643
MUC5B	**rs35705950**	0.003	0.0128	0.056	0.2504	0.085	0.345	0.107	0.3605	0.978	0.956	0.0221
SLC2A5	**rs2478868**	0.300	0.0128	0.291	0.2504	0.369	0.345	0.375	0.3605	0.7440	0.553	0.2560
JAK1	**rs12046291**	0.126	0.0128	0.451	0.2504	0.758	0.345	0.710	0.3605	0.9790	0.959	0.0207
AK5	**rs71658797**	0.004	0.0128	0.049	0.2504	0.017	0.345	0.086	0.3605	0.6480	0.419	0.3520
EFNA4	**rs114301457**	0	0.0128	0.001	0.2504	0.002	0.345	0.008	0.3605	0.6710	0.450	0.3290
TRIM46	**rs7528026**	0.003	0.0128	0.030	0.2504	0.018	0.345	0.020	0.3605	0.7040	0.495	0.2960
THBS3	**rs41264915**	0.262	0.0128	0.075	0.2504	0.115	0.345	0.132	0.3605	0.8220	0.676	0.1780
HCN3	**rs11264349**	0.282	0.0128	0.307	0.2504	1028	0.345	0.281	0.3605	0.4270	0.182	0.5730
BCL11A	**rs1123573**	0.362	0.0128	0.241	0.2504	0.293	0.345	0.387	0.3605	0.1610	0.0258	0.8390
-	**rs17713054**	0.004	0.0128	0.043	0.2504	0.301	0.345	0.081	0.3605	0.6250	0.390	0.3750
NXPE3	**rs11706494**	0.300	0.0128	0.285	0.2504	0.569	0.345	0.331	0.3605	0.4870	0.237	0.5130
PLSCR1	**rs343314**	0.098	0.0128	0.066	0.2504	0.033	0.345	0.076	0.3605	0.7340	0.538	0.2660
LOC105374536	**rs7664615**	0.477	0.0128	0.218	0.2504	0.681	0.345	0.173	0.3605	0.1500	0.0225	0.8500
ARHGEF38	**rs72670002**	0.002	0.0128	0.053	0.2504	0.015	0.345	0.061	0.3605	0.6330	0.4000	0.3670
-	**rs1073165**	0.203	0.0128	0.376	0.2504	0.433	0.345	0.403	0.3605	0.9810	0.9630	0.0186
IRF1-AS1	**rs2269821**	0.452	0.0128	0.148	0.2504	0.390	0.345	0.138	0.3605	0.5880	0.3460	0.4120
CCHCR1	**rs111837807**	0.057	0.0128	0.137	0.2504	0.112	0.345	0.112	0.3605	0.7880	0.6210	0.2120
LTA	**rs2071590**	0.249	0.0128	0.367	0.2504	0.476	0.345	0.364	0.3605	0.8500	0.7220	0.1500
-	**rs2858305**	0.319	0.0128	0.207	0.2504	0.456	0.345	0.341	0.3605	0.3210	0.1030	0.6790
-	**rs41435745**	0.089	0.0128	0.202	0.2504	0.488	0.345	0.032	0.3605	0.3610	0.1300	0.6390
HIP1	**rs1179620**	0.554	0.0128	0.360	0.2504	0.726	0.345	0.428	0.3605	0.0153	0.00023	0.9850
ZKSCAN1	**rs2897075**	0.098	0.0128	0.435	0.2504	0.874	0.345	0.386	0.3605	0.7560	0.5720	0.2440
RAB2A	**rs13276831**	0.349	0.0128	0.375	0.2504	1090	0.345	0.378	0.3605	0.4270	0.1820	0.5730
IFNA10	**rs28368148**	0.001	0.0128	0.006	0.2504	0.005	0.345	0.013	0.3605	0.7790	0.6070	0.2210
SFTPD	**rs721917**	0.599	0.0128	0.621	0.2504	0.678	0.345	0.580	0.3605	0.2960	0.08780	0.7040
ELF5	**rs61882275**	0.192	0.0128	0.408	0.2504	0.929	0.345	0.376	0.3605	0.6520	0.4250	0.3480
OAS1	**rs2660**	0.027	0.0128	0.205	0.2504	0.542	0.345	0.345	0.3605	0.8770	0.770	0.1230
-	**rs11614702**	0.148	0.0128	0.278	0.2504	0.622	0.345	0.474	0.3605	0.8760	0.7680	0.1240
ATP11A	**rs12585036**	0.062	0.0128	0.148	0.2504	0.457	0.345	0.215	0.3605	0.7280	0.5290	0.2720
SLC22A31	**rs117169628**	0.006	0.0128	0.115	0.2504	0.114	0.345	0.161	0.3605	0.9580	0.9190	0.0416
-	**rs12941811**	0.271	0.0128	0.341	0.2504	0.731	0.345	0.422	0.3605	0.6730	0.4540	0.3270
KANSL1	**rs8080583**	0.334	0.0128	0.192	0.2504	0.062	0.345	0.247	0.3605	0.7190	0.516	0.2810
-	**rs77534576**	0.002	0.0128	0.066	0.2504	0.094	0.345	0.034	0.3605	0.7190	0.517	0.2810
-	**rs142770866**	0.003	0.0128	0.048	0.2504	0.013	0.345	0.085	0.3605	0.6190	0.384	0.3810
FUT2	**rs516246**	0.489	0.0128	0.341	0.2504	0.285	0.345	0.441	0.3605	0.6240	0.389	0.3760
-	**rs35463555**	0.187	0.0128	0.372	0.2504	0.517	0.345	0.322	0.3605	0.7840	0.614	0.2160
CASC20	**rs2326788**	0.261	0.0128	0.486	0.2504	1150	0.345	0.386	0.3605	0.4270	0.183	0.5730
IFNAR2	**rs188401375**	0	0.0128	0.013	0.2504	0.003	0.345	0.022	0.3605	0.6260	0.392	0.3740
IFNAR2	**rs9636867**	0.190	0.0128	0.451	0.2504	1098	0.345	0.332	0.3605	0.4270	0.1830	0.5730
IL10RB	**rs8178521**	0.016	0.0128	0.245	0.2504	0.385	0.345	0.247	0.3605	0.9120	0.8320	0.0877
LOC105372790	**rs76608815**	0.006	0.0128	0.157	0.2504	0.364	0.345	0.097	0.3605	0.6670	0.4460	0.3330
TMPRSS2	**rs915823**	0.126	0.0128	0.117	0.2504	0.352	0.345	0.204	0.3605	0.6190	0.3830	0.3810
ACE2	**rs35697037**	0.266	0.0128	0.385	0.2504	0.654	0.345	0.384	0.3605	0.6930	0.4810	0.3070

(-) No annotation; AFR: African population; AMR: American population; EUR: European population; SAS: South Asian population; EAS: East Asian population.

Supplementary Table S2. SNPs that correlated with COVID-19 mortality in different populations

GENE	SNP ID	Freq. AFR	Mor AFR	Freq. AMR	Mor AMR	Freq. EAS+SAS	Mor EAS+SAS	Freq. EUR	Mor EUR	R	R^2	*P*_valor
STXBP5-AS1	**rs117928001**	0.003	0.0257	0.012	0.4276	0.026	0.1752	0.046	0.32	0.392	0.154	0.608
LOC105373891	**rs34011564**	0.123	0.0257	0.225	0.4276	0.208	0.1752	0.237	0.32	0.873	0.763	0.127
CPQ	**rs4735444**	0.368	0.0257	0.271	0.4276	0.938	0.1752	0.208	0.32	-0.382	0.146	0.618
-	**rs117217714**	0	0.0257	0.006	0.4276	0.008	0.1752	0.006	0.32	0.63	0.397	0.37
-	**rs2176724**	0.411	0.0257	0.102	0.4276	0.093	0.1752	0.11	0.32	-0.79	0.623	0.21
PCDH15	**rs9804218**	0.461	0.0257	0.514	0.4276	0.612	0.1752	0.693	0.32	0.369	0.136	0.631
CLUAP1	**rs2301762**	0	0.0257	0.071	0.4276	0.208	0.1752	0.052	0.32	0.106	0.0113	0.894
C3	**rs2547438**	0.042	0.0257	0.205	0.4276	0.332	0.1752	0.257	0.32	0.479	0.23	0.521
C3	**rs2250656**	0.184	0.0257	0.255	0.4276	0.48	0.1752	0.297	0.32	0.0688	0.00474	0.931
THBD	**rs1042580**	0.201	0.0257	0.291	0.4276	0.683	0.1752	0.368	0.32	0.0103	0.000107	0.99
CFH	**rs800292**	0.791	0.0257	0.419	0.4276	0.75	0.1752	0.26	0.32	-0.831	0.691	0.169
CFHR1	**rs414628**	0.326	0.0257	0.507	0.4276	0.897	0.1752	0.443	0.32	0.0679	0.00461	0.932
-	**rs73060484**	0.115	0.0257	0.236	0.4276	0.369	0.1752	0.077	0.32	0.0603	0.00363	0.94
-	**rs77578623**	0.114	0.0257	0.236	0.4276	0.372	0.1752	0.078	0.32	0.0613	0.00375	0.939
-	**rs74417002**	0.067	0.0257	0.024	0.4276	0.033	0.1752	0.026	0.32	-0.902	0.814	0.0978
-	**rs73070529**	0.218	0.0257	0.236	0.4276	0.281	0.1752	0.058	0.32	-0.269	0.0726	0.731
LINC01827	**rs113892140**	0.256	0.0257	0.238	0.4276	0.279	0.1752	0.046	0.32	-0.396	0.157	0.604
DNAH7	**rs183712207**	0.001	0.0257	0.009	0.4276	0	0.1752	0.009	0.32	0.871	0.758	0.129
DNAH7	**rs191631470**	0.001	0.0257	0.009	0.4276	0	0.1752	0.014	0.32	0.76	0.577	0.24
STBP5	**rs116898161**	0.002	0.0257	0.013	0.4276	0.026	0.1752	0.041	0.32	0.435	0.19	0.565
-	**rs55986907**	0.123	0.0257	0.337	0.4276	0.533	0.1752	0.284	0.32	0.324	0.105	0.676
CPQ	**rs1431889**	0.299	0.0257	0.262	0.4276	0.938	0.1752	0.209	0.32	-0.301	0.0905	0.699
CPQ	**rs2874140**	0.419	0.0257	0.287	0.4276	0.955	0.1752	0.21	0.32	-0.429	0.184	0.571
CPQ	**rs7007951**	0.348	0.0257	0.277	0.4276	0.935	0.1752	0.2	0.32	-0.353	0.125	0.647
CPQ	**rs920576**	0.322	0.0257	0.284	0.4276	0.961	0.1752	0.221	0.32	-0.305	0.0929	0.695
-	**rs60811869**	0.03	0.0257	0.019	0.4276	0.019	0.1752	0.03	0.32	-0.425	0.18	0.575
LZTFL1	**rs10490770**	0.004	0.0257	0.043	0.4276	0.301	0.1752	0.081	0.32	-0.0657	0.00432	0.934
-	**rs115679256**	0.002	0.0257	0.02	0.4276	0.01	0.1752	0.046	0.32	0.655	0.43	0.345
LOC107986082	**rs17763742**	0.004	0.0257	0.042	0.4276	0.306	0.1752	0.081	0.32	-0.0715	0.00511	0.929
FYCO1	**rs41289622**	0.005	0.0257	0.059	0.4276	0.365	0.1752	0.122	0.32	-0.0277	0.000769	0.972
LOC105377067	**rs115102354**	0.002	0.0257	0.019	0.4276	0.024	0.1752	0.048	0.32	0.571	0.326	0.429
-	**rs10813976**	0.11	0.0257	0.192	0.4276	0.432	0.1752	0.196	0.32	0.0386	0.00149	0.961
ARHGAP27	**rs1230082**	0.53	0.0257	0.373	0.4276	0.918	0.1752	0.303	0.32	-0.505	0.255	0.495
-	**rs77127536**	0.174	0.0257	0.141	0.4276	0.494	0.1752	0.181	0.32	-0.294	0.0867	0.706
IFNAR2	**rs17860169**	0.194	0.0257	0.451	0.4276	1.098	0.1752	0.333	0.32	-0.236	0.0557	0.764

FYCO1	**rs35477280**	0.005	0.0257	0.058	0.4276	0.378	0.1752	0.12	0.32	-0.04	0.0016	0.96
SLC6A20	**rs2271616**	0.011	0.0257	0.108	0.4276	0.183	0.1752	0.135	0.32	0.476	0.227	0.524
OAS1	**rs10774671**	0.64	0.0257	0.274	0.4276	0.545	0.1752	0.352	0.32	-0.99	0.979	0.0105
RAVER1	**rs74956615**	0.002	0.0257	0.026	0.4276	0.02	0.1752	0.03	0.32	0.891	0.793	0.109
TYK2	**rs34536443**	0.002	0.0257	0.02	0.4276	0.006	0.1752	0.029	0.32	0.827	0.683	0.173
PLEKHA4	**rs4801778**	0.16	0.0257	0.105	0.4276	0.166	0.1752	0.192	0.32	-0.427	0.183	0.573
PPP1R15A	**rs11541192**	0.247	0.0257	0.102	0.4276	0.185	0.1752	0.185	0.32	-0.927	0.859	0.0734
DPP9	**rs2109069**	0.196	0.0257	0.219	0.4276	0.326	0.1752	0.321	0.32	0.189	0.0355	0.811
FOXP4-AS1	**rs1886814**	0.083	0.0257	0.197	0.4276	0.494	0.1752	0.043	0.32	-0.0659	0.00435	0.934
FOXP4-AS1	**rs7741164**	0.089	0.0257	0.2	0.4276	0.488	0.1752	0.032	0.32	-0.0779	0.00607	0.922
KANSL1	**rs1819040**	0.062	0.0257	0.16	0.4276	0.065	0.1752	0.242	0.32	0.739	0.546	0.261
CD209	**rs4804803**	0.445	0.0257	0.157	0.4276	0.258	0.1752	0.216	0.32	-0.954	0.91	0.046
IFNAR2	**rs13050728**	0.194	0.0257	0.451	0.4276	1.095	0.1752	0.333	0.32	-0.236	0.0557	0.764
OAS1	**rs4767027**	0.011	0.0257	0.203	0.4276	0.538	0.1752	0.345	0.32	0.271	0.0732	0.729
DPP9	**rs12610495**	0.128	0.0257	0.203	0.4276	0.315	0.1752	0.294	0.32	0.364	0.132	0.636
TYK2	**rs11085727**	0.094	0.0257	0.258	0.4276	0.741	0.1752	0.27	0.32	0.0394	0.00155	0.961
HLA-DPA1	**rs2071351**	0.604	0.0257	0.281	0.4276	1.049	0.1752	0.186	0.32	-0.236	0.0559	0.764
ACE2	**rs190509934**	0.006	0.0257	0.006	0.4276	0.033	0.1752	0.003	0.32	-0.26	0.0677	0.74
MUC5B	**rs35705950**	0.003	0.0257	0.056	0.4276	0.085	0.1752	0.107	0.32	0.578	0.334	0.422
SLC2A5	**rs2478868**	0.300	0.0257	0.291	0.4276	0.369	0.1752	0.375	0.32	0.00982	0.0000964	0.990
JAK1	**rs12046291**	0.126	0.0257	0.451	0.4276	0.758	0.1752	0.710	0.32	0.46800	0.219	0.532
AK5	**rs71658797**	0.004	0.0257	0.049	0.4276	0.017	0.1752	0.086	0.32	0.75900	0.576	0.241
EFNA4	**rs114301457**	0	0.0257	0.001	0.4276	0.002	0.1752	0.008	0.32	0.38700	0.150	0.613
TRIM46	**rs7528026**	0.003	0.0257	0.030	0.4276	0.018	0.1752	0.020	0.32	0.96200	0.925	0.0382
THBS3	**rs41264915**	0.262	0.0257	0.075	0.4276	0.115	0.1752	0.132	0.32	0.87800	0.772	0.122
HCN3	**rs11264349**	0.282	0.0257	0.307	0.4276	1028	0.1752	0.281	0.32	0.23600	0.0558	0.764
BCL11A	**rs1123573**	0.362	0.0257	0.241	0.4276	0.293	0.1752	0.387	0.32	0.48000	0.230	0.520
-	**rs17713054**	0.004	0.0257	0.043	0.4276	0.301	0.1752	0.081	0.32	0.06570	0.00432	0.934
NXPE3	**rs11706494**	0.300	0.0257	0.285	0.4276	0.569	0.1752	0.331	0.32	0.24300	0.0588	0.757
PLSCR1	**rs343314**	0.098	0.0257	0.066	0.4276	0.033	0.1752	0.076	0.32	0.27500	0.0754	0.725
LOC105374536	**rs7664615**	0.477	0.0257	0.218	0.4276	0.681	0.1752	0.173	0.32	0.70000	0.491	0.300
ARHGEF38	**rs72670002**	0.002	0.0257	0.053	0.4276	0.015	0.1752	0.061	0.32	0.91700	0.841	0.0828
-	**rs1073165**	0.203	0.0257	0.376	0.4276	0.433	0.1752	0.403	0.32	0.65200	0.425	0.348
IRF1-AS1	**rs2269821**	0.452	0.0257	0.148	0.4276	0.390	0.1752	0.138	0.32	0.94000	0.883	0.0605
CCHCR1	**rs111837807**	0.057	0.0257	0.137	0.4276	0.112	0.1752	0.112	0.32	0.92500	0.855	0.0751
LTA	**rs2071590**	0.249	0.0257	0.367	0.4276	0.476	0.1752	0.364	0.32	0.36900	0.136	0.631
-	**rs2858305**	0.319	0.0257	0.207	0.4276	0.456	0.1752	0.341	0.32	0.52300	0.274	0.477
-	**rs41435745**	0.089	0.0257	0.202	0.4276	0.488	0.1752	0.032	0.32	0.07430	0.00553	0.926
HIP1	**rs1179620**	0.554	0.0257	0.360	0.4276	0.726	0.1752	0.428	0.32	0.68800	0.473	0.312
ZKSCAN1	**rs2897075**	0.098	0.0257	0.435	0.4276	0.874	0.1752	0.386	0.32	0.23800	0.0567	0.762

RAB2A	**rs13276831**	0.349	0.0257	0.375	0.4276	1090	0.1752	0.378	0.32	0.23600	0.0558	0.764
IFNA10	**rs28368148**	0.001	0.0257	0.006	0.4276	0.005	0.1752	0.013	0.32	0.64900	0.421	0.351
SFTPD	**rs721917**	0.599	0.0257	0.621	0.4276	0.678	0.1752	0.580	0.32	0.10200	0.0105	0.898
ELF5	**rs61882275**	0.192	0.0257	0.408	0.4276	0.929	0.1752	0.376	0.32	0.06480	0.00420	0.935
OAS1	**rs2660**	0.027	0.0257	0.205	0.4276	0.542	0.1752	0.345	0.32	0.24800	0.0616	0.752
-	**rs11614702**	0.148	0.0257	0.278	0.4276	0.622	0.1752	0.474	0.32	0.20400	0.0417	0.796
ATP11A	**rs12585036**	0.062	0.0257	0.148	0.4276	0.457	0.1752	0.215	0.32	0.05170	0.00267	0.948
SLC22A31	**rs117169628**	0.006	0.0257	0.115	0.4276	0.114	0.1752	0.161	0.32	0.78100	0.609	0.219
-	**rs12941811**	0.271	0.0257	0.341	0.4276	0.731	0.1752	0.422	0.32	0.02480	0.000615	0.9750
KANSL1	**rs8080583**	0.334	0.0257	0.192	0.4276	0.062	0.1752	0.247	0.32	0.29100	0.0849	0.709
-	**rs77534576**	0.002	0.0257	0.066	0.4276	0.094	0.1752	0.034	0.32	0.43800	0.192	0.562
-	**rs142770866**	0.003	0.0257	0.048	0.4276	0.013	0.1752	0.085	0.32	0.75500	0.571	0.245
FUT2	**rs516246**	0.489	0.0257	0.341	0.4276	0.285	0.1752	0.441	0.32	0.40100	0.161	0.599
-	**rs35463555**	0.187	0.0257	0.372	0.4276	0.517	0.1752	0.322	0.32	0.36400	0.132	0.636
CASC20	**rs2326788**	0.261	0.0257	0.486	0.4276	1150	0.1752	0.386	0.32	0.23600	0.0557	0.764
IFNAR2	**rs188401375**	0	0.0257	0.013	0.4276	0.003	0.1752	0.022	0.32	0.78300	0.613	0.217
IFNAR2	**rs9636867**	0.190	0.0257	0.451	0.4276	1098	0.1752	0.332	0.32	0.23600	0.0557	0.764
IL10RB	**rs8178521**	0.016	0.0257	0.245	0.4276	0.385	0.1752	0.247	0.32	0.49800	0.248	0.502
LOC105372790	**rs76608815**	0.006	0.0257	0.157	0.4276	0.364	0.1752	0.097	0.32	0.17700	0.0315	0.823
TMPRSS2	**rs915823**	0.126	0.0257	0.117	0.4276	0.352	0.1752	0.204	0.32	0.16200	0.0263	0.838
ACE2	**rs35697037**	0.266	0.0257	0.385	0.4276	0.654	0.1752	0.384	0.32	0.09770	0.00955	0.902

(-) No annotation; AFR: African population; AMR: American population; EUR: European population; SAS: South Asian population; EAS: East Asian population.

Supplementary Table S3. 25 studies included in the review.

ARTICLE	CITATION
Genetic variants are identified to increase risk of COVID-19 related mortality from UK Biobank data	Hu, J., Li, C., Wang, S. *et al.* Genetic variants are identified to increase risk of COVID-19 related mortality from UK Biobank data. *Hum Genomics* **15**, 10 (2021). https://doi.org/10.1186/s40246-021-00306-7 [1]
Whole-genome sequencing reveals host factors underlying critical COVID-19	Kousathanas, A., Pairo-Castineira, E., Rawlik, K. *et al.* Whole-genome sequencing reveals host factors underlying critical COVID-19. *Nature* **607**, 97–103 (2022). https://doi.org/10.1038/s41586-022-04576-6 [7]

GWAS and meta-analysis identifies 49 genetic variants underlying critical COVID-19.	Pairo-Castineira, E., Rawlik, K., Bretherick, A.D. *et al.* GWAS and meta-analysis identifies 49 genetic variants underlying critical COVID-19. *Nature* **617**, 764–768 (2023). https://doi.org/10.1038/s41586-023-06034-3 [13]
Mapping the human genetic architecture of COVID-19.	COVID-19 Host Genetics Initiative. Mapping the human genetic architecture of COVID-19. *Nature* **600**, 472–477 (2021). https://doi.org/10.1038/s41586-021-03767-x. [14]
Genome-wide association studies of COVID-19: Connecting the dots	Ferreira LC, Gomes CEM, Rodrigues-Neto JF, Jeronimo SMB. Genome-wide association studies of COVID-19: Connecting the dots. *Infect Genet Evol.* 2022 Dec; 106:105379. doi: 10.1016/j.meegid.2022.105379. [16]
Genetic mechanisms of critical illness in COVID-19	Pairo-Castineira, E., Clohisey, S., Klaric, L. *et al.* Genetic mechanisms of critical illness in COVID-19. *Nature* **591**, 92–98 (2021). https://doi.org/10.1038/s41586-020-03065-y [20]
Genome-wide analysis provides genetic evidence that ACE2 influences COVID-19 risk and yields risk scores associated with severe disease.	Horowitz, J.E., Kosmicki, J.A., Damask, A. *et al.* Genome-wide analysis provides genetic evidence that ACE2 influences COVID-19 risk and yields risk scores associated with severe disease. *Nat Genet* **54**, 382–392 (2022). https://doi.org/10.1038/s41588-021-01006-7 [29]
Genome-wide association study of COVID-19 severity among the Chinese population	Li, Y., Ke, Y., Xia, X. *et al.* Genome-wide association study of COVID-19 severity among the Chinese population. *Cell Discov* **7**, 76 (2021). https://doi.org/10.1038/s41421-021-00318-6 [30]
Host Genetic Variants Potentially Associated With SARS-CoV-2: A Multi-Population Analysis	Smatti MK, Al-Sarraj YA, Albagha O, Yassine HM. Host Genetic Variants Potentially Associated With SARS-CoV-2: A Multi-Population Analysis. *Front Genet*. 2020 Oct 2;11:578523. doi: 10.3389/fgene.2020.578523. [31]
Novel genes and sex differences in COVID-19 severity	Cruz R, Diz-de Almeida S, López de Heredia M, Quintela I, *et al*. Novel genes and sex differences in COVID-19 severity. *Hum Mol Genet*. 2022 Nov 10;31(22):3789-3806. doi: 10.1093/hmg/ddac132. [32]

Host genetic determinants of COVID-19 susceptibility and severity: A systematic review and meta-analysis.	Eshetie S, Jullian P, Benyamin B, Lee SH. Host genetic determinants of COVID-19 susceptibility and severity: A systematic review and meta-analysis. *Rev Med Virol*. 2023 Sep;33(5):e2466. doi: 10.1002/rmv.2466. [33]
Host genetic factors of COVID-19 susceptibility and disease severity in a Thai population	Chamnanphon, M., Pongpanich, M., Suttichet, T.B. *et al.* Host genetic factors of COVID-19 susceptibility and disease severity in a Thai population. *J Hum Genet* **67**, 295–301 (2022). https://doi.org/10.1038/s10038-021-01009-6 [34]
Identification of genetic loci jointly influencing COVID-19 and coronary heart diseases	Wang S, Peng H, Chen F, Liu C, Zheng Q, Wang M, Wang J, Yu H, Xue E, Chen X, Wang X, Fan M, Qin X, Wu Y, Li J, Ye Y, Chen D, Hu Y, Wu T. Identification of genetic loci jointly influencing COVID-19 and coronary heart diseases. *Hum Genomics.* 2023 Nov 14;17(1):101. doi: 10.1186/s40246-023-00547-8. [35]
Genetic prediction of ICU hospitalization and mortality in COVID-19 patients using artificial neural networks	Asteris PG, Gavriilaki E, Touloumenidou T, Koravou EE, Koutra M, *et al.* Genetic prediction of ICU hospitalization and mortality in COVID-19 patients using artificial neural networks. *J Cell Mol Med.* 2022 Mar;26(5):1445-1455. doi: 10.1111/jcmm.17098. [36]
Detailed stratified GWAS analysis for severe COVID-19 in four European populations	Degenhardt F, Ellinghaus D, Juzenas S, Lerga-Jaso J, *et al*. Detailed stratified GWAS analysis for severe COVID-19 in four European populations. *Hum Mol Genet.* 2022 Nov 28;31(23):3945-3966. doi: 10.1093/hmg/ddac158. [37]
Targeted screening of genetic associations with COVID-19 susceptibility and severity	Li P, Ke Y, Shen W, Shi S, Wang Y, Lin K, Guo X, Wang C, Zhang Y, Zhao Z. Targeted screening of genetic associations with COVID-19 susceptibility and severity. *Front Genet.* 2022 Nov 30;13:1073880. doi: 10.3389/fgene.2022.1073880. [38]
Shared genetic etiology between idiopathic pulmonary fibrosis and COVID-19 severity	Fadista J, Kraven LM, Karjalainen J, Andrews SJ, Geller F; COVID-19 Host Genetics Initiative; Baillie JK, Wain LV, Jenkins RG, Feenstra B. Shared genetic etiology between idiopathic pulmonary fibrosis and COVID-19 severity. *EBioMedicine*. 2021 Mar;

	65:103277. doi: 10.1016/j.ebiom.2021.103277. [39]
Integrative omics provide biological and clinical insights into acute respiratory distress syndrome	Du M, Garcia JGN, Christie JD, Xin J, Cai G, Meyer NJ, Zhu Z, Yuan Q, Zhang Z, Su L, Shen S, Dong X, Li H, Hutchinson JN, Tejera P, Lin X, Wang M, Chen F, Christiani DC. Integrative omics provide biological and clinical insights into acute respiratory distress syndrome. *Intensive Care Med*. 2021 Jul;47(7):761-771. doi: 10.1007/s00134-021-06410-5. [40]
Genetic justification of severe COVID-19 using a rigorous algorithm	Gavriilaki E, Asteris PG, Touloumenidou T, Koravou EE, Koutra M, Papayanni PG, Karali V, Papalexandri A, Varelas C, Chatzopoulou F, Chatzidimitriou M, Chatzidimitriou D, Veleni A, Grigoriadis S, Rapti E, Chloros D, Kioumis I, Kaimakamis E, Bitzani M, Boumpas D, Tsantes A, Sotiropoulos D, Sakellari I, Kalantzis IG, Parastatidis ST, Koopialipoor M, Cavaleri L, Armaghani DJ, Papadopoulou A, Brodsky RA, Kokoris S, Anagnostopoulos A. Genetic justification of severe COVID-19 using a rigorous algorithm. *Clin Immunol*. 2021 May;226:108726. doi: 10.1016/j.clim.2021.108726. [41]
Analysis of Plasma Proteins Involved in Inflammation, Immune Response/Complement System, and Blood Coagulation upon Admission of COVID-19 Patients to Hospital May Help to Predict the Prognosis of the Disease	di Flora DC, Dionizio A, Pereira HABS, Garbieri TF, Grizzo LT, Dionisio TJ, Leite AL, Silva-Costa LC, Buzalaf NR, Reis FN, Pereira VBR, Rosa DMC, Dos Santos CF, Buzalaf MAR. Analysis of Plasma Proteins Involved in Inflammation, Immune Response/Complement System, and Blood Coagulation upon Admission of COVID-19 Patients to Hospital May Help to Predict the Prognosis of the Disease. *Cells.* 2023 Jun 10;12(12):1601. doi: 10.3390/cells12121601. [42]
Host genetic polymorphisms involved in long-term symptoms of COVID-19	Udomsinprasert W, Nontawong N, Saengsiwaritt W, Panthan B, Jiaranai P, Thongchompoo N, Santon S, Runcharoen C, Sensorn I, Jittikoon J, Chaikledkaew U, Chantratita W. Host genetic polymorphisms involved in long-term symptoms of COVID-19. *Emerg Microbes Infect*. 2023 Dec;12(2):2239952. doi: 10.1080/22221751.2023.2239952. [43]
A MUC5B Gene Polymorphism, rs35705950-T, Confers Protective Effects Against COVID-19 Hospitalization but Not Severe Disease or Mortality	Verma A, Minnier J, Wan ES, Huffman JE, Gao L, Joseph J, Ho YL, Wu WC, Cho K, Gorman BR, Rajeevan N, Pyarajan S, Garcon H, Meigs JB, Sun YV, Reaven PD, McGeary JE, Suzuki A, Gelernter J,

	Lynch JA, Petersen JM, Zekavat SM, Natarajan P, Dalal S, Jhala DN, Arjomandi M, Gatsby E, Lynch KE, Bonomo RA, Freiberg M, Pathak GA, Zhou JJ, Donskey CJ, Madduri RK, Wells QS, Huang RDL, Polimanti R, Chang KM, Liao KP, Tsao PS, Wilson PWF, Hung AM, O'Donnell CJ, Gaziano JM, Hauger RL, Iyengar SK, Luoh SW; Million Veteran Program COVID-19 Science Initiative. A *MUC5B* Gene Polymorphism, rs35705950-T, Confers Protective Effects Against COVID-19 Hospitalization but Not Severe Disease or Mortality. *Am J Respir Crit Care Med.* 2022 Nov 15;206(10):1220-1229. doi: 10.1164/rccm.202109-2166OC. [44]
Host genetic factors determining COVID-19 susceptibility and severity	Velavan TP, Pallerla SR, Rüter J, Augustin Y, Kremsner PG, Krishna S, Meyer CG. Host genetic factors determining COVID-19 susceptibility and severity. *EBioMedicine.* 2021 Oct; 72:103629. doi: 10.1016/j.ebiom.2021.103629. [45]
A first update on mapping the human genetic architecture of COVID-19	COVID-19 Host Genetics Initiative. A first update on mapping the human genetic architecture of COVID-19. *Nature* **608**, E1–E10 (2022). https://doi.org/10.1038/s41586-022-04826-7 [46]
Multi-ancestry fine mapping implicates OAS1 splicing in risk of severe COVID-19	Huffman, J.E., Butler-Laporte, G., Khan, A. *et al.* Multi-ancestry fine mapping implicates *OAS1* splicing in risk of severe COVID-19. *Nat Genet* **54**, 125–127 (2022). https://doi.org/10.1038/s41588-021-00996-8 [47]
